# Testicular Germ Cell Tumor Tissue Biomarker Analysis: A Comparison of Human Protein Atlas and Individual Testicular Germ Cell Tumor Component Immunohistochemistry

**DOI:** 10.3390/cells12141841

**Published:** 2023-07-13

**Authors:** Jure Krasic, Lucija Skara Abramovic, Marta Himelreich Peric, Vedran Vanjorek, Marko Gangur, Dragana Zovko, Marina Malnar, Silvija Masic, Alma Demirovic, Bernardica Juric, Monika Ulamec, Marijana Coric, Davor Jezek, Tomislav Kulis, Nino Sincic

**Affiliations:** 1 Croatian Institute for Brain Research, School of Medicine, University of Zagreb, 10000 Zagreb, Croatia; 2 Centre of Excellence for Reproductive and Regenerative Medicine, School of Medicine, University of Zagreb, 10000 Zagreb, Croatia; 3 Department of Virology, Croatian Institute of Public Health, 10000 Zagreb, Croatia; 4 Health Centre Zagreb–West, 10000 Zagreb, Croatia; 5 Ljudevit Jurak Clinical Department of Pathology and Cytology, University Clinical Hospital Center Sestre Milosrdnice, 10000 Zagreb, Croatia; 6 School of Dental Medicine, University of Zagreb, 10000 Zagreb, Croatia; 7 Department of Pathology, School of Medicine, University of Zagreb, 10000 Zagreb, Croatia; 8 Department of Pathology and Cytology, University Hospital Centre Zagreb, 10000 Zagreb, Croatia; 9 Department of Histology and Embryology, School of Medicine, University of Zagreb, 10000 Zagreb, Croatia; 10 Department of Urology, University Hospital Centre Zagreb, 10000 Zagreb, Croatia; 11 Department of Urology, School of Medicine, University of Zagreb, 10000 Zagreb, Croatia; 12 Department of Biology, School of Medicine, University of Zagreb, 10000 Zagreb, Croatia

**Keywords:** testicular germ cell tumors, immunohistochemistry, histology, human protein atlas, digital pathology, biomarkers, pathology, pathohistology, IHC, healthy controls

## Abstract

The accurate management of testicular germ cell tumors (TGCTs) depends on identifying the individual histological tumor components. Currently available data on protein expression in TGCTs are limited. The human protein atlas (HPA) is a comprehensive resource presenting the expression and localization of proteins across tissue types and diseases. In this study, we have compared the data from the HPA with our in-house immunohistochemistry on core TGCT diagnostic genes to test reliability and potential biomarker genes. We have compared the protein expression of 15 genes in TGCT patients and non-neoplastic testicles with the data from the HPA. Protein expression was converted into diagnostic positivity. Our study discovered discrepancies in three of the six core TGCT diagnostic genes, *POU5F1*, *KIT* and *SOX17* in HPA. *DPPA3*, *CALCA* and *TDGF1* were presented as potential novel TGCT biomarkers. *MGMT* was confirmed while *RASSF1* and *PRSS21* were identified as biomarkers of healthy testicular tissue. Finally, *SALL4*, *SOX17*, *RASSF1* and *PRSS21* dysregulation in the surrounding testicular tissue with complete preserved spermatogenesis of TGCT patients was detected, a potential early sign of neoplastic transformation. We highlight the importance of a multidisciplinary collaborative approach to fully understand the protein landscape of human testis and its pathologies.

## 1. Introduction

Testicular germ cell tumors (TGCTs) are the most common neoplasm in males aged between 15 and 44 years [1,2]. TGCTs have had a rising global incidence of over 70% during the past 20 years, with the highest predicted rise in eastern and southern Europe [3,4]. The reason behind their rising incidence has not been fully understood, but is assumed to be a combination of environmental, inherited and epigenetic factors [5,6]. Their incidence is highest in developed Western countries and among people of European origin (with Norway having the highest incidence world-wide), in which they account for over 60% of all male malignancies between ages 20 and 40 [1,3].

TGCTs are developmental cancers that recapitulate several phenomena related to germ cell and embryonic development [7]. According to the WHO guidelines, they are separated into those that are germ cell neoplasia in situ (GCNIS)-derived and those that are not. GCNIS-derived TGCTs make up around 95% of all TGCT cases and in this study we have focused on them [4,8]. They are a highly complex group of tumors with heterogenous histology and can be subdivided into pure seminomas (SE) and non-seminomas (NSE), both occurring approximately equally [8,9]. NSE can be further divided according to their histological and cellular properties into embryonal carcinoma (EC), teratoma (TE), yolk sac tumor (YST) and choriocarcinoma (CH), with TE being the most chemo-resistant TGCT subtype [8,10]. Often, two or more TGCT histological elements are present in the tumor, which are then named mixed testicular germ cell tumors (MTGCTs). MTGCTs due to their histological heterogeneity are one of the most histologically complex tumors [11,12]. The 5-year survival rate of 95% shows that TGCTs are highly curable. If the disease is diagnosed in its early stages, the 5-year survival rate is 99% [13]. Despite the high cure rates overall, in NSE patients with poor prognosis, the 5-year survival rate decreases to 50%. Generally, patients with NSE have a worse prognosis than those with SE, with 20–40% of patients developing disease recurrence [14]. The most important risk factors of relapse in patients with stage I disease are the presence of lymphovascular invasion and the presence of EC [14,15].

TGCTs have a thorough staging system that combines clinical, pathological, radiological and serum tumor marker assessment, as staging plays a crucial role in the choice and sequence of treatment [15,16]. Hematoxylin and eosin stained slide (H&E) interpretation suffers from high rates of inter-observer variability, and using protein expression assay by immunohistochemistry (IHC) improves sensitivity [14]. IHC is the gold standard in pathohistological TGCT diagnostics due to its necessity in determining histological components within the TGCT, which is crucial for determining prognosis and patient management. The histological heterogeneity and complexity within TGCTs allow for possible morphological overlaps and problematic areas for accurate diagnosis, for which IHC analysis is crucial [12,17,18]. The presence of GCNIS can only be confidently ascertained with IHC analysis, as the light microscopy of H&E slides underestimates its presence [19,20]. The final diagnosis of TGCTs is only possible after radical orchidectomy has been performed and the excised testis has undergone pathohistological examination. Tissue biopsies are not performed in suspected cases of TGCT due to the risks of potential complications. While promising biomarkers are emerging for early diagnostic purposes, such as mir-371-3p, they cannot identify the histological subtypes of TGCT present or differentiate between necrosis/fibrosis and TE [21].

The complexity of TGCTs staging, coupled with their relative rarity, leads to issues in clinical practices. Pathologists may only see a few cases of TGCT per year, which can lead to increased rates of misdiagnosis. Discrepancies in assessment between general pathology departments and centers with expertise in TGCT have already been reported, with most disagreements being in interpreting histological subtypes (SE or NSE). The reproducibility of parameter reporting by pathologists in TGCT is far from ideal, with studies reporting possible discrepancies in up to 31% of patients, with 9% of patients receiving alterations in prognosis and 6.5% having an alteration in their predicted impact on therapy [22,23]. Dedicated centers and professionals are required for accurate staging and further research on TGCT [24]. In some countries, like Canada, it has been regulated that all histological specimens should be examined by a pathologist experienced in TGCT [22].

Molecular biology and genomic approaches have led to the discovery of several hundred cancer testis genes, most of which are identified using gene expression on the mRNA level (mRNA expression described further in the text). However, the gene expression on the protein level (protein expression described further in the text) in the testis is still unclear, and the human protein atlas (HPA) has been used as a reference to determine the testicular proteome [25]. The HPA is a free-to-use and open-access platform with the aim of mapping all the human proteins in the body. It has contributed to thousands of publications and assists in antibody validation, selection and in silico protein analysis [26].

In this study, we have compared the TGCT protein expression data available from the HPA with results produced by our group. We have focused on NSE, in particular on MTGCT, as most issues in TGCT diagnostics come from trying to diagnose its histological components correctly. SE components included in this research were only present as part of MTGCT. Candidate genes were selected based on either being core TGCT diagnostic markers routinely used in the clinic (*POU5F1*, *NANOG*, *SOX2*, *SOX17*, *KIT*, *SALL4*), which were used to compare the reliability of HPA and our group, or by being identified as potential TGCT biomarkers on DNA methylation or mRNA levels with little to no research existing on a spatial tissue level (*RASSF1*, *MGMT*, *HOXA9*, *TDGF1*, *MAGEC2*, *KITLG*, *DPPA3*, *CALCA*, *PRSS21* and 5mC).

## 2. Materials and Methods

### 2.1. HPA Analysis

The protein expression data of the target genes were collected from the human protein atlas version 22.0 [26]. Briefly, staining intensity (0—no staining, 1—low intensity, 2—medium intensity and 3—high intensity) and quantity (0–0%, 1–<25%, 2–75%–25%, 3–>75%) for every enrolled patient/volunteer were obtained for the analyzed components, and every antibody was investigated. The immunoreactivity score (IRS) was obtained by multiplying the intensity and quantity of each analyzed sample.

The diagnostic positivity of the analyzed samples was determined using the following cut-off: all slides with an intensity of 0 and 1 were declared negative while slides with an intensity of 2 and above were declared positive.

All available antibodies per investigated gene were included in the analysis. HPA data were accessed on the 15th of February 2023. 17 TGCT samples were analyzed. Seminiferous tubules and Leydig cells were analyzed in the surrounding healthy tissue of patients with neoplastic disease and in non-neoplastic patients.

### 2.2. In-House Analysis

#### 2.2.1. Patients

Altogether, formalin-fixed paraffin-embedded (FFPE) blocks from 58 patients were collected from University Hospital Centre Zagreb and the University Hospital Centre Sestre Milosrdnice paraffin tissue archive. A total of 38 TGCT patients with a confirmed diagnosis of NSE were recruited with their FFPE blocks being collected for further analysis. FFPE blocks with non-neoplastic testicular tissue samples from 20 patients were collected. FFPE non-neoplastic testicular tissue samples were taken from patients admitted to the hospital due to non-neoplastic issues such as testicular hemorrhaging, hydrocele, and testicular torsion with healthy tissue with preserved spermatogenesis, without inflammatory changes or hemorrhage, which was selected for the study.

#### 2.2.2. Ethical Statement

Information about the study was given to all the participants who gave written consent. The study was conducted according to the Declaration of Helsinki. The Ethics Committee of the School of Medicine University of Zagreb (protocol code 380-59-10106-23-111/37, 641-01/23-02/01), University Hospital Centre Sestre Milosrdnice (protocol code, number: EP-18327/17-3) and University Hospital Centre Zagreb (protocol code 8.1-18/72-2, number: 02/21 AG) approved the collection and manipulation of all tissue samples.

#### 2.2.3. H&E Analysis

**Figure 1:**
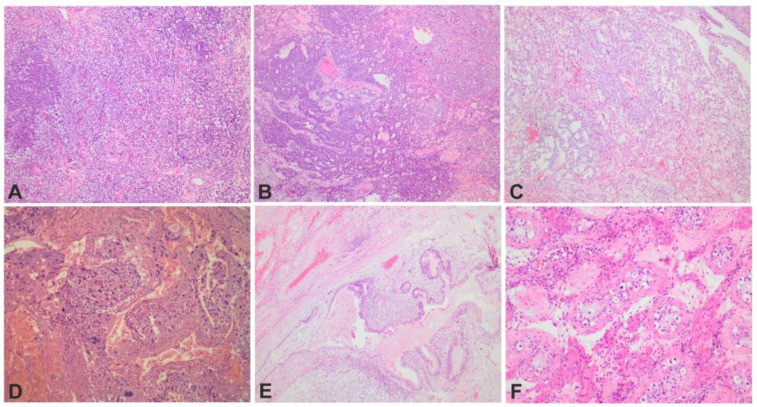
HE histology of different components found in mixed TGCT; (**A**) seminoma (HE × 100); (**B**) embryonal carcinoma (HE × 100); (**C**) yolk sac tumor (HE × 100), (**D**) choriocarcinoma (HE × 100); (**E**) teratoma (HE × 100); (**F**) seminiferous tubules with germ cell neoplasia in situ and Leydig cells in between them (HE × 200).

**Figure 2:**
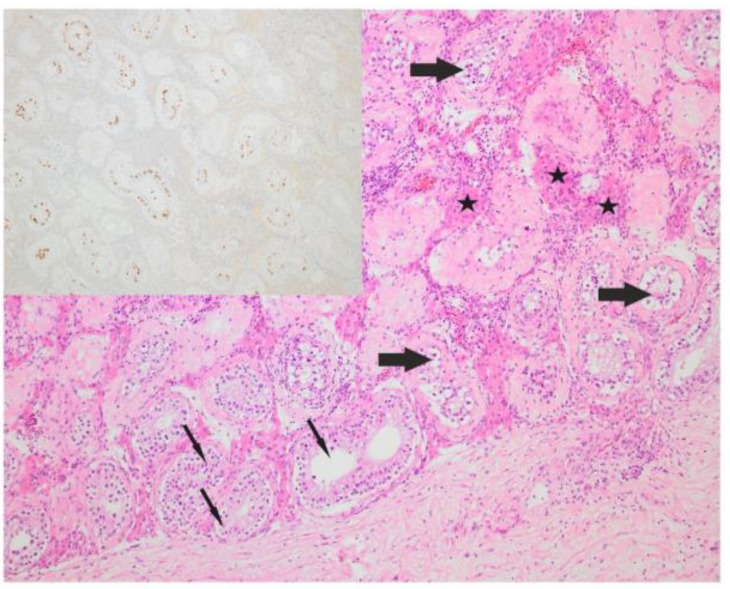
Seminiferous tubules with germ cell neoplasia in situ (thick arrow) and preserved spermatogenesis (thin arrow), Leydig cells in between tubules (asterisk) (HE × 200). In the left upper corner is the immunohistochemistry of *POU5F1*, showing a positive reaction in the in situ component (*POU5F1* × 100).

FFPE blocks were sectioned at 4 µm. H&E stained slides were examined by two general pathologists (D.A, J.B) and three uropathologists (U.M., M.S. and C.M.) to confirm the diagnosis and identify subtypes present and the presence of GCNIS and spermatogenesis. All disagreements were resolved with the team’s joint decision. The components analyzed in this study were seminiferous tubules with preserved spermatogenesis (ST), Leydig cells (LEY), GCNIS, EC, TE, YST, SE and CH (Figure 1). Seminiferous tubules and Leydig cells were analyzed both in TGCT patients’ surrounding healthy testicular tissue with complete preserved spermatogenesis (SHT) and in non-neoplastic testicular tissue from patients with no TGCT and with complete preserved spermatogenesis (NNT) (Figure 2).

#### 2.2.4. IHC

IHC was performed according to standardized protocols, of which a detailed description is described in available publications [27,28,29].

Prepared FFPE microscopic slides were deparaffinized, cleared in xylene and rehydrated in a series of decreasing alcohol solutions ending in TBS. Heat-induced epitope retrieval was performed using a vegetable steamer. The slides were blocked with 5% goat serum after which they were incubated overnight at 4 °C with primary antibody diluted in 1% BSA/TBS/0.1% Tween-20 (antibody details in Supplementary S1). Slides were then rinsed with TBS, incubated with 3% H_2_O_2_ to block endogenous peroxidase activity and rinsed again with TBS. The next step involved the application of a secondary antibody (Dako REAL EnVi-sion Detection System, K5007, Agilent Technologies, Santa Clara, CA, USA) and incubation at 37 °C for 1 h, followed by a serial TBS wash three times for 5 min. Subsequently, the samples were incubated for 6 min in DAB (3,3-diaminobenzidine-tetrahydrochloride) (Dako REAL EnVision Detection System, K5007, Agilent Technologies, Santa Clara, CA, USA). Slides were counterstained with hematoxylin, embedded and analyzed using the Olympus BX51 microscope (Olympus, Tokyo, Japan). Appropriate positive and negative controls for each antibody were used.

#### 2.2.5. IHC Analysis

H score was calculated by multiplying the percentage of positive cells (within the analyzed component) with the intensity pattern of the staining [30]. The signal was analyzed in the nucleus, cytoplasm or membrane, depending on the antibody involved. Staining intensity (0—no staining, 1—low intensity, 2—medium intensity and 3—high intensity) and staining percentage (0–100%) were analyzed in each of the previously listed components of the analyzed testicular tissue separately, by two general pathologists (D.A and J.B) and three uropathologists (U.M., M.S. and C.M.). All disagreements were resolved by a joint committee.

Results are shown as individual analyzed components, and as TGCT as a whole tumor to match how HPA presents its results.

Diagnostic positivity was determined as follows: all slides with an intensity of 1 were considered negative. Slides with an intensity of 2 and above and positive staining of 10% and above were considered diagnostically positive [31].

## 3. Results

### 3.1. Patient Data

The data of patients and controls used by HPA are shown in Table 1.

The relevant patients’ clinicopathological data are shown in Table 2.

### 3.2. HPA Analysis

**Figure 3:**
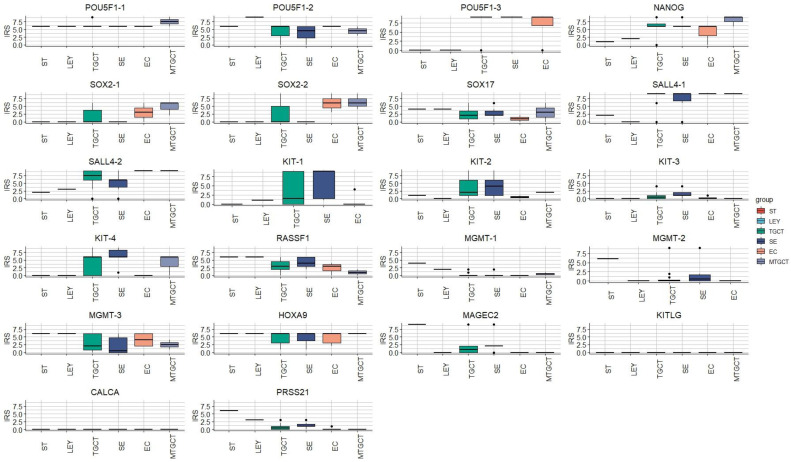
Protein expression data, obtained from HPA in TGCT and healthy testicular tissue samples. ST—seminiferous tubules with preserved spermatogenesis, LEY—Leydig cells, TGCT—testicular germ cell tumor, SE—seminoma, EC—embryonal carcinoma, MTGCT—mixed testicular germ cell tumor.

Protein expression data obtained from HPA across the analyzed components are shown in Figure 3. Seminiferous tubules and Leydig cells from the surrounding healthy testicular tissue with complete preserved spermatogenesis of TGCT patients and non-neoplastic patients were pooled together into a single seminiferous tubule or Leydig cell group.

**Figure 4:**
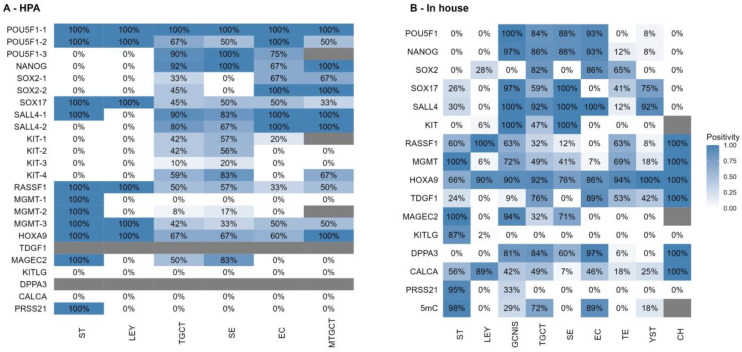
(**A**) HPA; Diagnostic positivity of core diagnostic and potential biomarker genes, from samples obtained from the HPA in TGCT and healthy testicular tissue samples. (**B**) In-house; Diagnostic positivity of core diagnostic and potential biomarker genes, from the patient cohort recruited for this study in TGCT and healthy testicular tissue samples. ST—seminiferous tubules with preserved spermatogenesis, LEY—Leydig cells, GCNIS—germ cell neoplasia in situ, TGCT—testicular germ cell tumor, SE—seminoma, EC—embryonal carcinoma, TE—teratoma, YST—yolk sac tumor, MTGCT—mixed testicular germ cell tumor, CH—choriocarcinoma.

Diagnostic positivity calculated from the samples from the HPA is shown in Figure 4A—HPA.

### 3.3. IHC Analysis

**Figure 5:**
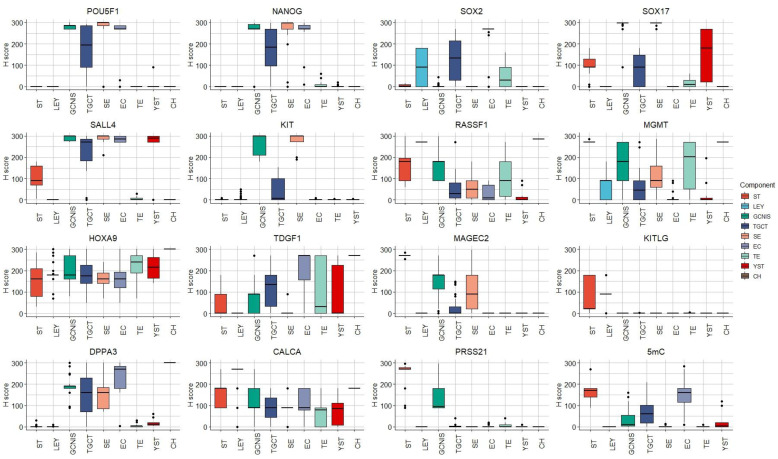
Protein expression data, obtained by our research group in TGCT and healthy testicular tissue samples. ST—seminiferous tubules with preserved spermatogenesis, LEY—Leydig cells, GCNIS—germ cell neoplasia in situ, TGCT—testicular germ cell tumor, SE—seminoma, EC—embryonal carcinoma, TE—teratoma, YST—yolk sac tumor, CH—choriocarcinoma.

Protein expression data produced by our research group across the analyzed components are shown in Figure 5. Seminiferous tubules and Leydig cells from the surrounding healthy testicular tissue with complete preserved spermatogenesis of TGCT patients and non-neoplastic patients were pooled together into a single seminiferous tubule or Leydig cell group.

The calculated diagnostic positivity from the patient cohort recruited for this study is shown in Figure 4B—In house.

### 3.4. TGCT Patients vs. Non-Neoplastic Patients Testicular Tissue

**Figure 6:**
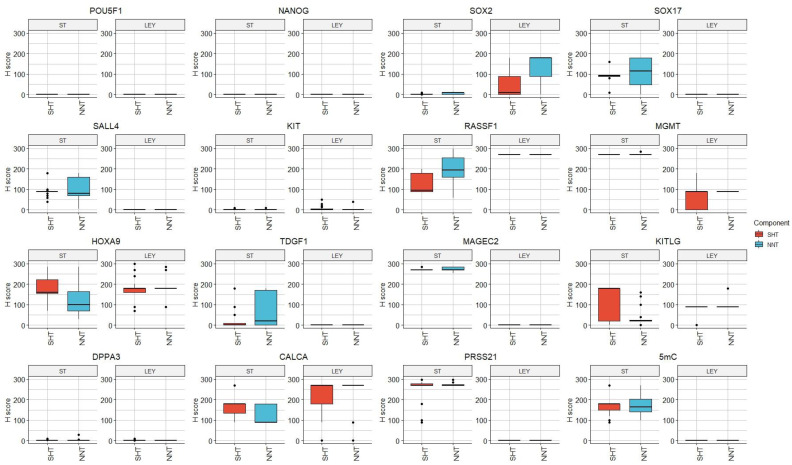
Protein expression data obtained by our research group; a comparison between the healthy surrounding testicular tissue of patients with testicular germ cell tumors (SHTs) and the healthy testicular tissue of patients with no malignant disease (NNT). ST—seminiferous tubules, LEY—Leydig cells.

Protein expression data, produced by our research group, of seminiferous tubules and Leydig cells from the surrounding healthy testicular tissue with complete preserved spermatogenesis of TGCT patients and non-neoplastic patients, are shown in Figure 6.

**Figure 7:**
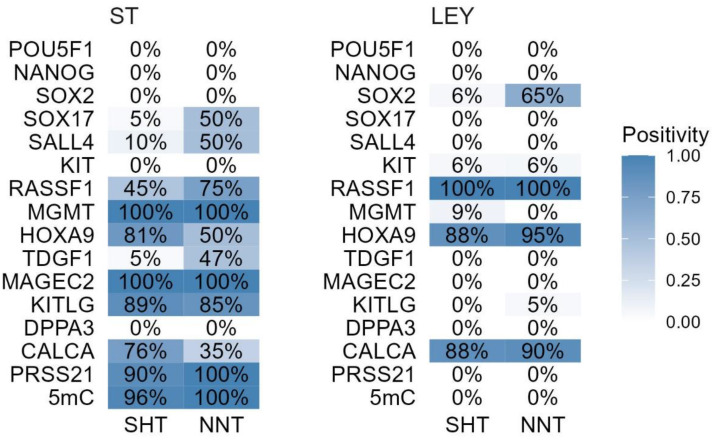
Diagnostic positivity of core diagnostic and potential biomarker genes, a comparison between the healthy surrounding testicular tissue of patients with testicular germ cell tumors (SHTs) and the healthy testicular tissue of patients with no malignant disease (NNT). ST—seminiferous tubules, LEY—Leydig cells.

The calculated diagnostic positivity in the surrounding healthy testicular tissue with complete preserved spermatogenesis of TGCT patients and non-neoplastic patients is shown in Figure 7.

### 3.5. Comparison of Results

We have analyzed the genes *POU5F1*, *NANOG*, *SOX2*, *SOX17*, *SALL4* and *KIT* as a quality control benchmark since the expression on the protein level of these genes is well investigated and due to their routine use in pathohistological diagnostics [32].

The HPA data of *POU5F1* protein expression have shown high positivity across all analyzed components. mRNA expression, however, has not shown expression in healthy testicles, while its expression was increased in TCGA data (Supplementary S2). The results of our protein expression analysis for *POU5F1* correspond to the available literature, with GCNIS, EC and SE having high expression and no expression in other components, seminiferous tubules or Leydig cells [12,33].

HPA data of *NANOG* protein expression have shown positivity in TGCT across SE, EC and MTGCT, corresponding to the mRNA expression data from the available databases. This shows TGCT as a whole positive for *NANOG*. The results of our protein expression analysis for *NANOG* are similar and correspond to the available literature [34,35]. No positivity in healthy testicular tissue and low to no positivity in TE, YST and CH was detected.

HPA data of *SOX2* protein expression have shown similar results across two antibodies, around 40% positivity in TGCT and no positivity in healthy testicular tissue. When looked at individually, the positivity was found in the EC and MTGCT samples, and not in the SE samples. mRNA expression confirms no expression in healthy testicular tissue and expression in TGCT. The results of our protein expression analysis for *SOX2* have shown positivity in EC and TE, corresponding to the available literature [36].

HPA data of *SOX17* protein expression have shown positivity in both seminiferous tubules and Leydig cells and half of the TGCT samples. Regarding individual TGCT patients, SE, EC and MTGCT are about equally positive for *SOX17*. At the same time, mRNA expression shows some expression in healthy testicular tissue as well as in individual TGCT. However, *SOX17* is used in TGCT diagnostics routinely to distinguish SE from EC and is uniformly positive in the SE samples and negative in EC samples. The results of our protein expression analysis for *SOX17* correspond to these results, as we have confirmed expression in some seminiferous tubules and almost all of the GCNIS and SE samples. The detected positivity in TE and YST has been described before in the literature [37]. Most importantly, all EC samples were negative.

HPA data of *SALL4* protein expression across two analyzed antibodies have shown conflicting results for seminiferous tubules, with it being uniformly positive in one and negative in the other. As for TGCT tissues, it is positive in most TGCT, the only negative samples being SE. mRNA expression is in line with that, showing some expression in healthy testicular tissue and expression in TGCT. The results of our protein expression analysis for *SALL4* correspond to this, showing positivity in a subset of seminiferous tubules, no positivity in Leydig cells and an almost uniform positivity across GCNIS and TGCT, except for TE and CH [38,39].

HPA data of *KIT* protein expression are uniform across four antibodies on healthy testicular tissue showing no positivity in seminiferous tubules or Leydig cells. As for TGCT samples, it shows low-to-medium positivity, with most of the positive samples being SE (around 50% of SE) and most EC or MTGCT being negative. mRNA expression is in line with that, showing expression in healthy testicular tissue and TGCT. The results of our protein expression analysis for *KIT* correspond to this, with positivity being uniform in GCNIS and SE, and the other components being negative [40]. Again, these HPA data are misleading because *KIT* is a universal diagnostic biomarker of SE and GCNIS.

Overall, the HPA data of *POU5F1*, *SOX17* and *KIT* show significant deviations from commonly accepted knowledge of their positivity in TGCT.

*RASSF1* protein expression analysis in the HPA dataset has shown uniform positivity in seminiferous tubules and Leydig cells, decreasing positivity in TGCT, with lower positivity in EC than in SE. mRNA expression is in line with that, showing expression in healthy testicular tissue and lower expression in TGCT. The results of our protein expression analysis for *RASSF1* confirms this, with positivity decreasing from healthy testicular tissue to GCNIS to SE to YST and finally to EC. TE and CH have a higher positivity and expression, which could in part explain their resistance to cisplatin; while, to our knowledge, no research exists on *RASSF1* at the protein level, we have found mRNA data from TGCT samples, and the research agrees that, in TGCT samples, an inactivation of *RASSF1* happens [41].

*MGMT* protein expression analysis in the HPA dataset across three antibodies has shown uniform positivity in seminiferous tubules and uniform negativity in two antibodies in Leydig cells. While one antibody has shown no *MGMT* expression in TGCT, the remaining two have shown a decreasing positivity in TGCT. mRNA expression is in line with that, showing expression in healthy testicular tissue and lower expression in TGCT. The results of our protein expression analysis for *MGMT* confirm the HPA analysis, with seminiferous tubules being positive, Leydig cells being negative and TGCT samples being increasingly negative, from GCNIS to SE to YST to EC, again with TE and CH being higher in positivity and expression than other TGCT samples. The existing literature aligns with both our results and those of the HPA [42].

*HOXA9* protein expression analysis in the HPA dataset has shown uniform positivity in seminiferous tubules and Leydig cells and decreased positivity in TGCT samples. mRNA expression has, however, detected no expression in healthy testicles or TGCT samples. The results of our protein expression analysis for *HOXA9* have shown most samples both in the healthy testicular tissue and TGCT samples as positive. However, SE and EC were less positive than YST, CH and TE. To our knowledge, no prior research has been conducted on TGCT using IHC for *RASSF1* or *HOXA9*.

*MAGEC2* protein expression analysis in the HPA dataset has shown uniform positivity in seminiferous tubules, and most of the SE samples were positive. mRNA expression has been detected in healthy testicles with a lesser expression in TGCT. The results of our protein expression analysis for *MAGEC2* are in line with this, with uniform positivity in seminiferous tubules, GCNIS and most of SE. The existing literature is in line with these results [43].

*KITLG* protein expression analysis in the HPA dataset has shown uniform negativity across all samples. mRNA expression has been detected in healthy testicles. The results of our protein expression analysis for *KITLG* align with this, with uniform positivity in seminiferous tubules and no expression in TGCT or GCNIS. The publications that have investigated *KITLG* report both overexpression in TGCT and lower expression than in healthy testicular tissue [44,45], while the research on mRNA expression in TGCT suggests lower expression of *KITLG*. Considering the uniformity of HPA analysis, mRNA analysis and our own research, we would argue that *KITLG* is not expressed in TGCT.

*TDGF1* protein expression analysis in the HPA dataset is not available. mRNA expression has detected expression in TGCT samples and no expression in healthy testicles. The results of our protein expression analysis for *TDGF1* have shown positivity in EC and lesser positivity in TE and YST, with some seminiferous tubules being positive and no expression in SE. The only article, to our knowledge, that investigated *TDGF1* in TGCT on IHC is mainly in accordance, except they detected the expression of *TDGF1* in SE as well [46]. However, they reported that they detected no mRNA expression in SE samples, which other articles have also reported and aligns with our results [46,47].

*CALCA* protein expression analysis in the HPA dataset is uniformly negative in healthy testicles and TGCT. mRNA expression has detected no expression in TGCT samples or healthy testicles. The results of our protein expression analysis for *CALCA* have shown high positivity in seminiferous tubules and Leydig cells and lesser positivity in GCNIS and EC, with progressively fewer positive samples in YST, TE and SE. To the best of our knowledge, this is the first research on the protein expression of *CALCA* in TGCT samples.

*DPPA3* protein expression analysis in the HPA dataset is not available. mRNA expression has detected no expression in healthy testicles and high expression in TGCT. The results of our protein expression analysis for *DPPA3* have shown no positivity in seminiferous tubules and Leydig cells and high positivity in GCNIS and EC, with less positive samples in SE and almost no positive samples in YST and TE. To the best of our knowledge, this is the first research on the protein expression of *DPPA3* in TGCT samples. However, research exists showing gene expression on the mRNA in SE and healthy testicles and it shows an increase in mRNA expression in half of the analyzed SE, which corresponds to our results [48].

*PRSS21* protein expression analysis in the HPA dataset has shown uniform positivity in seminiferous tubules and no positivity in Leydig cells or TGCT. mRNA expression has detected no expression in TGCT and high expression in healthy testicles. The results of our protein expression analysis for *PRSS21* have shown uniform positivity in seminiferous tubules and no positivity in Leydig cells, GCNIS or TGCT; while to the best of our knowledge this is the first study investigating *PRSS21* expression on the protein level, the available data on the mRNA level in TGCT confirm the inactivation of *PRSS21* [41].

Like the other DNA or protein modifications, 5 mC data are not shown in the HPA. Similar to previously published research, we confirm high positivity in seminiferous tubules, high positivity in EC, no positivity in SE and low positivity in GCNIS [49,50]. However, unlike them, we have detected no positivity in TE and low positivity in YST.

## 4. Discussion

The HPA available data on TGCT and testicular tissue are as follows: for IHC data, healthy testicular tissue is quantified as either seminiferous tubules or Leydig cells and is available from two or three volunteers per antibody analyzed, making statistical analysis impossible. TGCT patients were mainly identified as SE, with some EC and MTGCT patients present. A maximum of 12 TGCT samples were analyzed per antibody, with SE making up most of the analyzed samples. The small number of analyzed EC and MTGCT samples makes statistical analysis impossible.

TGCT patients’ data are presented in bulk per analyzed antibody, grouping all SE and NSE patients’ protein expressions. The information on histological subtypes is available only upon closer individual inspection of the slides and we have had to separate them as SE, EC or MTGCT ourselves. Patients with MTGCT were analyzed as bulk tumors, with no information on the protein expression in its components. No data exist for GCNIS or pure forms of YST, TE or CH. While this may not seem crucial at first glance, the clinical outcome for the patients depends on the TGCT components present, meaning that different histological components of TGCTs should be analyzed separately in any further research to accurately explore their protein landscape [32].

Another issue is how the HPA presents protein expression by conveying all analyzed antibodies for a protein as equally correct, regardless of commonly accepted clinical facts. mRNA expression data shown on HPA (from the HPA, GTEx, FANTOM5 and TCGA datasets) can be used as a guide to protein expression and in antibody selection. However, this opens more issues as mRNA data were generated from samples of non-neoplastic testicles and TGCT tissue and were analyzed as bulk (no information on seminiferous tubules or Leydig cells, only approximated instead as a percentage within the tissue) and no information even on histological subtype (SE or NSE). This in particular could be easily amended by allowing experts within the field to provide feedback on the individual antibodies.

Finally, while the HPA presents all its data as reactivity (a parameter of protein expression), it is not ideal for pathology or biomarker discovery. Diagnostic positivity as a value should be used in those instances as it has a minimum requirement for tissues to be declared positive or negative, and is how patients are routinely diagnosed in the clinic [31].

### 4.1. Healthy Controls

The subject of healthy controls has been controversial in TGCT diagnostics and has been handled in one of three possible ways. The first option is that a TGCT patient’s testicular tissue without apparent neoplastic changes and with present seminiferous tubules with complete preserved spermatogenesis is taken as “healthy”—SHT [51,52]. The second option is that testicular tissue from patients with non-neoplastic issues with present seminiferous tubules with complete preserved spermatogenesis can be provided by the urology departments and taken as “healthy”—NNT [36,50]. Finally, both types of samples can be combined into a control group, which is also performed by the HPA [42,53]. The surrounding healthy testicular tissue with complete preserved spermatogenesis from TGCT patients is most readily available as a control for TGCT studies and is what the pathologist will see contrasting the tumor tissue, or while searching for GCNIS. However, it is known that TGCT dysregulates the testis and its microenvironment [54,55]. The testicles of TGCT patients may also be affected by testicular dysgenesis syndrome, which is related to TGCT, meaning that their testicular tissue may already be dysregulated [56,57]. Non-neoplastic testicles provided by urology departments are usually biopsies from men suspected of infertility or cancer, and a large part of them have some other underlying condition (prostate cancer, testicular infarction, hydrocele or infertility). To the best of our knowledge, this is the first study to compare the surrounding healthy testicular tissue with complete preserved spermatogenesis of TGCT patients with non-neoplastic patients, analyzing both seminiferous tubules and Leydig cells.

The decreased positivity in the seminiferous tubules with complete preserved spermatogenesis of TGCT patients, compared to non-neoplastic patients, in *SALL4*, *SOX17*, *RASSF1* and *PRSS21* as well as increased *CALCA* positivity, could be a sign of dysregulated testicular environment due to TGCT or, perhaps, the result of the underlying testicular dysgenesis syndrome in the patients. *RASSF1*, along with its role in cell-cycle control, cellular adhesion, motility and apoptosis, has been identified as a tumor suppressor gene. It has been assumed that *RASSF1* inactivation may be among the first events of TGCT tumorigenesis [58]. *PRSS21* is assumed to act as a tumor suppressor gene whose inactivation plays an essential role in TGCT development [6]. *SALL4* is vital in mammalian germ cell development and regulating spermatogonial proliferation [59,60]. *SOX17* is involved in the regulation of apoptosis in germ cells and is found expressed during the maturation stages of spermatogenesis [37,61]. *CALCA* is the most potent neural peptide that dilates the blood vessels in the body. It is increased in the body’s cells following ischemia, hypoxia and oxygen-free radicals. After a period of prolonged increase, *CALCA* levels undergo marked reduction, as in patients with cryptorchidism [62]. This increase in *CALCA* expression may be a marker of pathological transformation in the testicular tissue of TGCT patients. This is reinforced by almost no difference in Leydig cells detected between the healthy testicular tissue with complete preserved spermatogenesis of TGCT patients and non-neoplastic patients, except for *SOX2* having lower positivity in TGCT patients, which could be due to Leydig cells having “scattered” *SOX2* positivity generally [63].

The detected discrepancy illustrates the difficulties of obtaining and adequately describing the protein landscape of the human testis and its pathologies. Assuming morphological normality and the presence of spermatogenesis as healthy masks potential malignant changes. In this study, we show the dysregulation of protein expression in the surrounding healthy testicular tissue with complete preserved spermatogenesis of TGCT patients for the first time with potential implications for TGCT patient management and testis sparing surgery decisions as well as future genetic and epigenetic studies using the selection of the control samples.

### 4.2. TGCT IHC Analysis

As mentioned before, the HPA data for *POU5F1*, *SOX17* and *KIT* have shown deviations from what is commonly accepted, and these are routinely used biomarkers in the diagnostic algorithm. In the case of *POU5F1*, in two of the three analyzed antibodies, STs were uniformly positive. *POU5F1* is used in routine clinical diagnostics of GCNIS precisely due to its uniform positivity in GCNIS, with the rare cases of positivity in gonads being in patients with disorders of sex development [18,33]. *SOX17* has shown equal positivity in SE and EC and *KIT* has shown medium positivity in SE, both of which go against commonly accepted facts in the pathology of TGCT. The reliability of our research group’s results has been confirmed by our results of the core TGCT diagnostic genes as they all align with the pathological guidelines [32,64]. This highlights the importance of interdisciplinary research teams incorporating specialized uropathologists.

While studies exploring protein expression for *MAGEC2*, *TDGF1*, *KITLG* and *MGMT* using IHC exist, for *RASSF1* and *PRSS21,* only mRNA expression and DNA methylation levels have been investigated. Finally, for *HOXA9*, *CALCA* and *DPPA3,* only DNA methylation was investigated in TGCT [65,66,67].

The HPA data and our results were, mainly, in line with *RASSF1*, *MGMT*, *HOXA9 MAGEC2* and *PRSS21*, with the main difference being our results showing the full scope of TGCT components. Again, this is especially relevant in MTGCT and GCNIS, in which the HPA did not separate individual histological components or did not quantify them. Our results confirm the potential of *MGMT* and, for the first time, show *RASSF1* and *PRSS21* as tissue biomarkers of normal testicular function whose inactivation is a crucial part of early TGCT tumorigenesis [6,58]. *MGMT* downregulation has also been shown in patients with azoospermia, solidifying its role as an integral gene for testicular function [68]. As for *CALCA*, *DPPA3* and *TDGF1,* we present novel results across all analyzed components. *CALCA* methylation was shown to be increased in NSE patients and correlated to refractory disease and poor clinical outcomes in TGCT [67]. More importantly, it has been shown that a prolonged increase in *CALCA* levels causes the subsequent reduction in *CALCA* levels associated with its pathological effect [62]. This could explain the reduced *CALCA* levels in TE and YST compared to EC. A hallmark of TGCTs is a state of conservation of primordial germ cell-lineage erasure of maternal and paternal genomic imprints, including in *DPPA3*. Previous studies have shown that the hypomethylation of *DPPA3* across all histological subtypes of TGCT is similar to the hypomethylation in primordial germ cells, which also all show protein expression of *DPPA3* [65]. In this research, however, we have shown an increase in *DPPA3* expression in SE and EC components and its loss in YST and TE. Our results are consistent with *DPPA3* being a marker of pluripotency and its inactivation during differentiation, similarly to how, in the healthy testis or somatic tissues, no *DPPA3* expression is present [48,69]. *TDGF1* is upregulated in testicular germ cells by activation of the Nodal pathway, which controls pluripotency and differentiation in embryonic stem cells. In this way, *TDGF1* and *DPPA3* are related to the fetal origin of TGCTs [65,70]. High levels of *TDGF1* expression are a sign of pluripotent cellular status, and have been shown to correlate to tumor invasiveness and the number of malignant cells [47]. While the expression of *TDGF1* is characteristic of EC and YST, we have also detected its expression in TE. As *TDGF1* has a regulatory function in the tumorigenicity of TGCTs [47], its expression in some TE could be of interest for further research. Finally, *KITLG* illustrates that genes exist in TGCT research without consensus and shows that original research is often sparse. We believe that our data and the data from HPA should reopen this field of discussion.

Specific subtypes of TGCT can morphologically overlap, such as areas with architecture typical of YST but with atypical cytological features, atypical areas within or adjacent to SE, which suggest transformation to EC, and the interface of EC and YST with cytological features unlike YST. These cases require IHC for proper diagnosis [32,71]. In our patient cohort, we have had multiple such instances, with the interface of YST and EC only being resolved with IHC analysis. Accurate diagnosis is essential for proper treatment selection, such as diagnosing the presence of EC within the TGCT [71]. All this makes knowing the protein landscape of TGCT histological components and the healthy testis essential for accurate TGCT management.

### 4.3. Study Highlights

Insufficient information available on protein expression in non-neoplastic testis compared to TGCT testis has made HPA in silico protein expression analysis increasingly popular in TGCT research [72,73,74]. As the usefulness and applications of HPA continue to increase, it would greatly benefit from including information on individual histological components of composite tumors, such as MTGCT, and the ability to receive feedback from other research groups on the antibodies used in protein investigation.

We have summarized the main findings of our study as follows:

1. The HPA is a useful online tool for exploration of gene expression on a protein level;
2. Due to the TGCT heterogeneity of histological components, bulk protein expression, as shown in the HPA, should be avoided;
3. Discrepancies in key TGCT diagnostic biomarkers were detected in the HPA;
4. *MAGEC2*, *HOXA9* and 5 mC were confirmed as potential TGCT biomarkers;
5. *DPPA3*, *CALCA* and *TDGF1* were identified as potential novel TGCT biomarkers;
6. *MGMT* was confirmed as a biomarker of healthy testicular tissue;
7. *RASSF1* and *PRSS21* were identified as biomarkers of healthy testicular tissue;
8. *SALL4*, *SOX17*, *RASSF1* and *PRSS21* dysregulation in the surrounding testicular tissue with complete preserved spermatogenesis was detected.

### 4.4. Limitations

A potential limitation of the current study is the relatively small number of enrolled patients, which reflects the relatively rare incidence of the disease. However, with patients and healthy controls being recruited from two of the largest hospital centers in Croatia, we are confident that the cohort used in this study has no single-center selection bias [15]. The cohort is a fully NSE patient cohort which also minimizes potential confounding variables and presents the landscape of MTGCT [64].

## 5. Conclusions

This research highlights the discrepancies in HPA data for genes used in the TGCT diagnostic algorithm. By analyzing protein expression in each component of the TGCT, we present a complete overview of the analyzed genes, confidently identifying *PRSS21*, *RASSF1* and *MGMT* as markers of the healthy testis. We also present novel protein expression results in TGCT for *TDGF1*, *CALCA* and *DPPA3*, unavailable in the HPA. We have, for the first time, identified the dysregulation of the genes *SALL4*, *SOX17*, *RASSF1* and *PRSS21* in the surrounding healthy testicular tissue of TGCT patients (with complete preserved spermatogenesis) with implications on genetic and epigenetic studies using it as control tissue. Finally, greater cooperation between different groups and platforms will facilitate greater progress in the understanding and management of TGCT.

## Figures and Tables

**Table 1:** The number of controls and patients visible on HPA, along with their diagnoses. TGCT—testicular germ cell tumor, SE—seminoma, EC—embryonal carcinoma, MTGCT—mixed testicular germ cell tumor.

HPA Data		
Controls	Testis	3
		
Diagnosis	TGCT	17
	SE	9
	EC	5
	MTGCT	3

**Table 2:** Patients’ clinicopathological data. TGCT—testicular germ cell tumor, TE—teratoma, EC—embryonal carcinoma, MTGCT—mixed testicular germ cell tumor, YST—yolk sac tumor, SE—seminoma, CH—choriocarcinoma, TNM—tumor category involving tumor size, lymph node involvement and metastatic spread.

Clinicopathological Data		
TGCT patients	38	
	
Age (median, range)	29.5 (18–45)	
	
TNM	T1	21
	T2	13
	T3	4
		
Stage	I	33
	II	2
	III	3
		
TGCT diagnosis	EC	10
	TE	2
	MTGCT	26
		
Components within MTGCT	EC	19
	TE	15
	YST	12
	SE	18
	CH	1

## Data Availability

The raw data supporting the conclusions of this article will be made available by the authors, without undue reservation.

## Supplementary Materials

The following supporting information can be downloaded at: https://www.mdpi.com/article/10.3390/cells12141841/s1, Supplementary S1: Antibodies used in IHC, Supplementary S2: HPA data on mRNA expression, Supplementary S3: HPA data on protein expression.
