# Supplementary material for: Testicular Germ Cell Tumor Tissue Biomarker Analysis: A Comparison of Human Protein Atlas and Individual Testicular Germ Cell Tumor Component Immunohistochemistry
Source: Cells. 2023 Jul 13;12(14):1841. doi: 10.3390/cells12141841 (PMC10378501; doi:10.3390/cells12141841)
Supplement: Supplementary file 1 [file cells-12-01841-s001.zip › Supplementary S1.pdf]

Antibody	Manufacturer	Cat. Number	HIER	Dilution	Secondary antibody	Positive control	Negative control
POU5F1	Abcam	ab109183	pH6	1:750	REAL EnVision Detection System, HRP/DAB, Rabbit/Mouse	seminoma	colorectal carcinoma
HOXA9	Atlas Antibodies	HPA061982	pH9	1:1300	REAL EnVision Detection System, HRP/DAB, Rabbit/Mouse	colon	kidney cancer
SOX2	Atlas Antibodies	AMAb91307	pH9	1:500	REAL EnVision Detection System, HRP/DAB, Rabbit/Mouse	embryonal carcinoma	muscle
NANOG	Abcam	ab106465	pH6	1:500	REAL EnVision Detection System, HRP/DAB, Rabbit/Mouse	seminoma	colon
PRSS21	Atlas Antibodies	HPA008477	pH9	1:100	REAL EnVision Detection System, HRP/DAB, Rabbit/Mouse	testis	colon
RASSF1	Atlas Antibodies	HPA040735	pH9	1:225	REAL EnVision Detection System, HRP/DAB, Rabbit/Mouse	colon	kidney cancer
SALL4	Atlas Antibodies	HPA015291	pH9	1:500	REAL EnVision Detection System, HRP/DAB, Rabbit/Mouse	testis	colon
MGMT	Atlas Antibodies	HPA069497	pH9	1:500	REAL EnVision Detection System, HRP/DAB, Rabbit/Mouse	colon	kidney cancer
TDGF1	Abcam	ab19917	pH6	1:550	REAL EnVision Detection System, HRP/DAB, Rabbit/Mouse	ovary	colon
DPPA3	Santa Cruz	sc-376862	pH9	1:225	REAL EnVision Detection System, HRP/DAB, Rabbit/Mouse	TGCT	colon
CALCA	Abcam	ab240922	pH9	1:600	REAL EnVision Detection System, HRP/DAB, Rabbit/Mouse	thyroid	testis
MAGEC2	Atlas Antibodies	HPA062230	pH6	1:500	REAL EnVision Detection System, HRP/DAB, Rabbit/Mouse	testis	liver
SOX17	Abcam	ab224637	pH9	1:1000	REAL EnVision Detection System, HRP/DAB, Rabbit/Mouse	seminoma	liver
KIT	Dako	A4502	En Vision Flex- Target retrival solution, high Ph	1:100	REAL EnVision Detection System, HRP/DAB, Rabbit/Mouse	ovary	colon
KITLG	Atlas Antibodies	HPA070395	pH9	1:500	REAL EnVision Detection System, HRP/DAB, Rabbit/Mouse	prostate cancer	colon
5mC	Abcam	(ab214727)	pH6	1000	REAL EnVision Detection System, HRP/DAB, Rabbit/Mouse		
