# Supplementary material for: Testicular Germ Cell Tumor Tissue Biomarker Analysis: A Comparison of Human Protein Atlas and Individual Testicular Germ Cell Tumor Component Immunohistochemistry
Source: Cells. 2023 Jul 13;12(14):1841. doi: 10.3390/cells12141841 (PMC10378501; doi:10.3390/cells12141841)
Supplement: Supplementary file 1 [file cells-12-01841-s001.zip › Supplementary S2.pdf]

Gene	Database	Value	
POU5F1	HPA	**1.3**	nTPM
	GTEX	0.8	nTPM
	FANTOM5	0	Scaled Tags Per Million
	TCGA	355.9	FPKM
NANOG	HPA	**0.9**	nTPM
	GTEX	0.2	nTPM
	FANTOM5	0	Scaled Tags Per Million
	TCGA	58.1	FPKM
SOX2	HPA	1.4	nTPM
	GTEX	**1.9**	nTPM
	FANTOM5	6.4	Scaled Tags Per Million
	TCGA	20.7	FPKM
SOX17	HPA	1.2	nTPM
	GTEX	**3.4**	nTPM
	FANTOM5	15.6	Scaled Tags Per Million
	TCGA	25.3	FPKM
KIT	HPA	**6.2**	nTPM
	GTEX	2.3	nTPM
	FANTOM5	13.6	Scaled Tags Per Million
	TCGA	28.6	FPKM
KITLG	HPA	**6.3**	nTPM
	GTEX	0.3	nTPM
	FANTOM5	36.8	Scaled Tags Per Million
	TCGA	2.2	FPKM
MAGEC2	HPA	**19.6**	nTPM
	GTEX	17.9	nTPM
	FANTOM5	27.4	Scaled Tags Per Million
	TCGA	6.7	FPKM
MGMT	HPA	9.9	nTPM
	GTEX	**15.2**	nTPM
	FANTOM5	25.7	Scaled Tags Per Million
	TCGA	5.3	FPKM
RASSF1	HPA	19.4	nTPM
	GTEX	**38.5**	nTPM
	FANTOM5	27.7	Scaled Tags Per Million
	TCGA	8	FPKM
PRSS21	HPA	27.1	nTPM
	GTEX	**84.6**	nTPM
	FANTOM5	23.1	Scaled Tags Per Million
	TCGA	2.2	FPKM

SALL4	HPA	3	nTPM
	GTEX	**3.9**	nTPM
	FANTOM5	3.4	Scaled Tags Per Million
	TCGA	22.7	FPKM
TDGF1	HPA	**0.5**	nTPM
	GTEX	0.4	nTPM
	FANTOM5	0.1	Scaled Tags Per Million
	TCGA	27.1	FPKM
DPPA3	HPA	**0.7**	nTPM
	GTEX	0.4	nTPM
	FANTOM5	0.1	Scaled Tags Per Million
	TCGA	339	FPKM
CALCA	HPA	**0.2**	nTPM
	GTEX	0.2	nTPM
	FANTOM5	0.2	Scaled Tags Per Million
	TCGA	0	FPKM
HOXA9	HPA	**0**	nTPM
	GTEX	0	nTPM
	FANTOM5	2	Scaled Tags Per Million
	TCGA	0.7	FPKM
